# Glycosylation and counter-glycosylation define an apple-insect chemical arms race

**DOI:** 10.1093/plphys/kiag308

**Published:** 2026-05-23

**Authors:** Marcella Teixeira, Praveen Khatri

**Affiliations:** Assistant Features Editor, Plant Physiology, American Society of Plant Biologists; Department of Plant Pathology, Washington State University, Pullman, WA 99163, United States; Assistant Features Editor, Plant Physiology, American Society of Plant Biologists; Department of Biology, University of Toronto at Mississauga, Mississauga, ON L5L 1C6, Canada

Over the past 100 million years, plants and herbivorous insects have coevolved together, resulting in a prolonged competition in which chemical defense often plays a decisive role (Labandeira et al. 1994). Unable to escape their attackers, plants have developed a sophisticated arsenal of chemical defenses, deploying diverse specialized metabolites like flavonoids, terpenoids, and alkaloids to minimize herbivore feeding and damage (Vasantha-Srinivasan et al. 2025). Among them, flavonoids have received special interest due to their chemical and biological diversity, being involved in different processes from plant development and stress adaptation to herbivore defense. Importantly, flavonoids are often covalently modified such as by glycosylation, and their biological activities can depend strongly on where they are stored and how they are mobilized within plant tissues.

In plant cells, most flavonoids are typically stored within vacuoles as glycosylated compounds instead of free aglycons, enhancing their stability and solubility (Zhu et al. 2024). This raises a central question: what is the role of glycosylation in defense mediated by flavonoids? Glycosides tend to be less harmful to herbivores, with toxicity unleashed upon hydrolysis into aglycons, halting herbivore-caused damage (Ferreira et al. 2018). Nevertheless, this framework is not universal. Recent studies in tea showed that quercetin glucosides per se can be effective anti-herbivore agents, whereas in apple, hydrolysis of dihydrochalcone glycoside phlorizin can also contribute to spider-mite resistance (Chen et al. 2024; Zhou et al. 2025). Moreover, plants employ various sugar donors for glycosylation, including glucose, galactose, xylose, rhamnose, and arabinose (Zhu et al. 2024). However, the contribution of these sugar moieties to herbivore resistance remains unexplored. With a rich repertoire of flavonoid glycosides, including quercetin derivatives and phlorizin, apple offers an ideal system to address the role of glycosylation in insect resistance.

Jasmonic acid (JA) is a central regulator of anti-herbivore defense, orchestrating the biosynthesis of specialized metabolites during plant stress. JA signaling promotes flavonoid biosynthesis, including flavonols such as quercetin, through the activation of MYB transcription factors (eg, MYB12) and downstream genes (Shen et al. 2022). Nevertheless, the mechanisms through which JA regulates flavonoid metabolism during herbivory remain unclear. Does herbivory selectively drive the production of quercetin glycosides or broad flavonoid defenses? Do different quercetin glycosides confer equivalent protection? How do herbivores metabolically react to these compounds?

In this issue of *Plant Physiology*, Xu et al. (2026) address these questions and demonstrate that quercetin glycosylation and hydrolysis are not merely biochemical modifications but key moves in an ongoing coevolutionary chess match between apple and its herbivores. Using transcriptome analysis of apple leaves attacked by *Helicoverpa armigera* or *Spodoptera litura*, the authors identified 1,833 differentially expressed genes (DEGs) shared between the 2 treatments, including those associated with biosynthesis of quercetin glycosides and JA. Consistently, treatments of apple leaves with methyl jasmonate (MeJA) enhanced expression of genes involved in quercetin glycosides biosynthesis and enhanced production of 5 quercetin glycosides, indicating that JA acts upstream of quercetin metabolic pathway.

To further dissect this regulation, the authors manipulated *MdGSTF9*, a glutathione S-transferase that reduces JA biosynthesis by conjugating the JA precursor oxylipin 12-oxo-phytodienoic acid (OPDA) with glutathione (GSH). Silencing *MdGSTF9* increased JA levels and promoted accumulation of 5 quercetin glycosides, resulting in reduced feeding preference by both herbivores. These findings establish a direct link between JA signaling and the production of specific flavonoid glycosides that contribute to herbivore deterrence.

One of the genes involved in accumulation of quercetin glycosides identified in the transcriptome analysis is *MdMYB22*, known to promote quercetin glycosides biosynthesis through activation of flavonol Synthase (FLS) (Wang et al. 2017). Overexpression of *MdMYB22* or *MdFLS* increased quercetin glycoside accumulation and reduced herbivore feeding, whereas silencing *MdFLS* had the opposite effect. Notably, manipulation of these genes did not alter JA levels, indicating that flavonoid glycosylation operates downstream of JA without feedback regulation.

Apple leaves produce 2 pairs of quercetin glycoside isomers. One of these pairs, quercetin-3-O-galactoside (Q3Gal) and quercetin-3-O-glucoside (Q3Glc), is biosynthesized by flavonol 3-O-galactosyltransferase (MdF3GalT) and flavonol 3-O-glucosyltransferase (MdF3GlcT), respectively. Xu et al. (2026) generated transgenic plants overexpressing or silencing *MdF3GalT* and *MdF3GlcT*. Manipulation of *MdF3GalT* led to altered Q3Gal levels, although no effects were observed in larvae feeding preference. In contrast, manipulation of *MdF3GlcT* resulted in altered Q3Glc levels, and its silencing caused lower Q3Glc and reduced preference by both *H. armigera* and *S. litura*. These results were confirmed with exogenous feeding assays, where both larvae preferred diets lacking Q3Glc, while Q3Gal had no effect on feeding preference, suggesting Q3Glc has anti-feeding activity.

This is a striking finding from the analysis of quercetin glycoside isomers. Genetic and biochemical analyses showed that Q3Glc, but not Q3Gal, is hydrolyzed by plant enzymes to release the toxic aglycone quercetin during herbivory. This selective hydrolysis reveals that glycosylation patterns determine not only metabolite identity but also defensive function.

The study further uncovered a remarkable counterstrategy employed by insects. While plant enzymes convert Q3Glc into quercetin, some herbivores, including *H. armigera* and *S. exigua*, can re-glycosylate quercetin to regenerate Q3Glc, thereby detoxifying the compound. In contrast, *S. litura* and *S. frugiperda* lack this capability and exhibit reduced growth when exposed to quercetin. This species-specific metabolic response highlights a previously unknown detoxification mechanism and underscores the coevolutionary dynamics shaping plant-herbivore interactions.

Together, these findings support a model in which herbivore attack activates JA signaling, leading to the accumulation of specific flavonoid glycosides such as Q3Glc. Upon feeding, plant enzymes hydrolyze Q3Glc to release toxic quercetin, deterring herbivores. In response, some insects counter this plant defense by re-glycosylating quercetin, effectively neutralizing its toxicity. Thus, glycosylation and counter-glycosylation form a metabolic battleground in which plants and insects continuously adapt to each other's strategies (Fig. 1).

**Figure 1:**
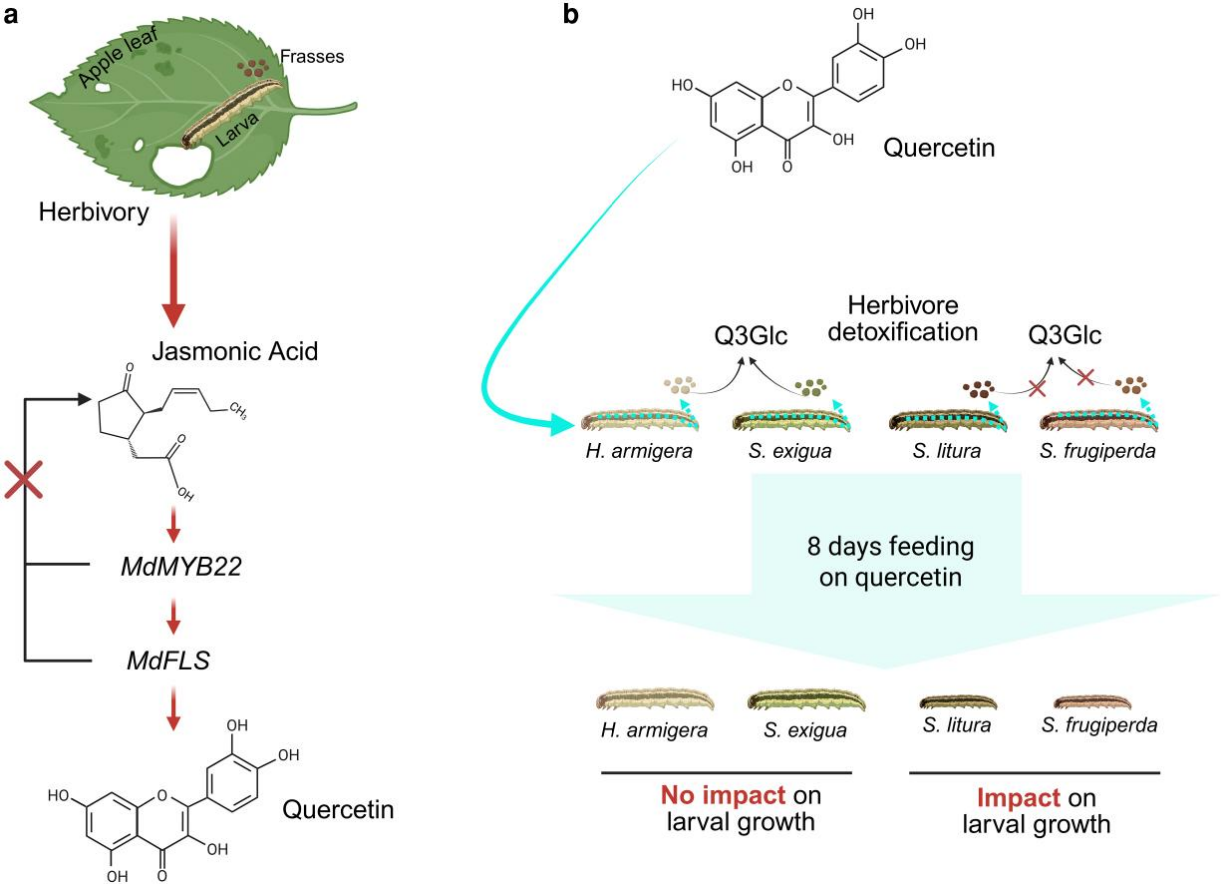
Model of jasmonate-regulated flavonoid glycosylation and insect counter-defense in apple. a) Herbivore attack induces JA signaling, which activates the transcription factor MdMYB22 and downstream FLS, promoting the accumulation of quercetin glycosides. These metabolites, particularly Q3Glc, are hydrolyzed by plant β-glucosidases during feeding to release the toxic aglycone quercetin. Flavonoid metabolism operates downstream of JA without feedback regulation. b) Some herbivores (*Helicoverpa armigera* and *Spodoptera exigua*) detoxify quercetin through re-glycosylation, whereas others (*Spodoptera litura* and *S. frugiperda*) lack this capacity and exhibit reduced growth. This ability was observed by detecting Q3Glc in frasses of *H. armigera* and *S. exigua* but not *S. litura* and *S. frugiperda*. This dynamic interplay highlights glycosylation and counter-glycosylation as key components of plant-herbivore coevolution. Created in BioRender. Teixeira, M. (2026) https://BioRender.com/xg2phte.

By identifying Q3Glc as a key determinant of herbivore resistance and defining the genetic components underlying its accumulation, the study by Xu et al. (2026) provides new opportunities to enhance natural defense in apple. Manipulating flavonoid glycosylation or hydrolysis pathways may offer a promising strategy for improving resistance against herbivores that lack effective detoxification mechanisms.

Recent related articles published in *Plant Physiology*:

Lu et al. (2025) showed that quercetin is a significant defense-related metabolite. The transcription factor CitPH4 induces the production of quercetin, and exogenous quercetin suppresses the growth of *Xanthomonas citri* subsp. *citri* (Xcc) and *Penicillium italicum*, major citrus pathogens.

Kundu et al. (2025) demonstrated that flavonoids and their derivatives participate in sorghum defense against pest fall armyworm (FAW; *Spodoptera frugiperda*), as higher flavonoid levels were linked to increased resistance following caterpillar attack.

## Data Availability

No data is generated in this study.
